# Mortality and Its Predictors in COVID-19 Patients With Pre-existing Interstitial Lung Disease

**DOI:** 10.7759/cureus.27759

**Published:** 2022-08-07

**Authors:** Naveen Dutt, Saumya Shishir, Nishant K Chauhan, Ramniwas Jalandra, Ashok kuwal, Pawan Garg, Deepak Kumar, Vikarn Vishwajeet, Amartya Chakraborti, Kunal Deokar, Shahir Asfahan, Avinash Babu, Pradeep bajad, Neeraj Gupta, Alkesh Khurana, Mahendra Kumar Garg

**Affiliations:** 1 Pulmonary and Critical Care Medicine, All India Institute of Medical Sciences, Jodhpur, Jodhpur, IND; 2 Diagnostic and Interventional Radiology, All India Institute of Medical Sciences, Jodhpur, Jodhpur, IND; 3 Internal Medicine, All India Institute of Medical Sciences, Jodhpur, Jodhpur, IND; 4 Pathology, All India Institute of Medical Sciences, Jodhpur, Jodhpur, IND; 5 Pulmonary and Critical Care Medicine, All India Institute of Medical Sciences, Rajkot, Rajkot, IND; 6 Pulmonary Medicine, Jawaharlal Nehru Medical College, Ajmer, IND; 7 Pulmonary and Critical Care Medicine, All India Institute of Medical Sciences, Bhopal, Bhopal, IND

**Keywords:** elevated d-dimer, interleukin (il)-6, mortality, interstitial lung disease, covid-19

## Abstract

Background

The data on the impact of coronavirus disease 2019 (COVID-19) on interstitial lung disease (ILD) is still limited. To the best of our knowledge, there has been no study from India to date to assess the impact of COVID-19 in patients with preexisting ILD. We undertook this study to assess the clinical outcome of ILD patients admitted to our hospital with COVID-19.

Methods

In this retrospective observational study, records of reverse transcription polymerase chain reaction (RT-PCR)-confirmed COVID-19 patients with preexisting ILD who were admitted to the hospital in the period from May 1, 2020, to April 30, 2021, were obtained from the hospital database. The clinical outcomes of the patients were recorded. Univariate analysis was performed to find relation between various predetermined risk factors for mortality and those with significant p values (p<0.05) were subjected to multiple logistic regression to determine independent risk factors.

Results

In our study of 28 patients, the overall mortality was 35.7%. On comparing the parameters associated with increased mortality, there was no effect of age, gender, comorbidities, type of ILD, CT thorax findings on diagnosis, use of corticosteroids and antifibrotics in the past, spirometric findings on mortality. On multivariate analysis, the significant parameters were interleukin 6 (IL-6), p=0.02, OR=1.020 (1.006-1.043) and D-dimer, p=0.04, OR=2.14 (5.55-1.14).

Conclusion

COVID-19 in patients with pre-existing ILD has a comparatively higher mortality. D-dimer and IL-6 are significant predictors of mortality in ILD patients infected with COVID-19.

## Introduction

Interstitial lung diseases (ILDs) include a broad range of acute and chronic diffuse lung disorders with both known and unidentified etiologies. They are distinguished by injury to alveoli followed by unregulated healing resulting in interstitial thickening. The disease manifests by gradual onset of symptoms like breathlessness on exertion, persistent non-productive cough, along with symptoms of underlying disease like connective tissue disease if present. The condition can advance to respiratory failure and eventually death if undiagnosed or untreated.

ILDs, most notably idiopathic pulmonary fibrosis (IPF), are characterized by acute exacerbation, defined as an acute, clinical deterioration or worsening of symptoms typically less than a month duration, new radiological findings of ground-glass and/or consolidation, not explained by heart failure or fluid overload. It can be triggered by infections, thoracic surgical procedures, and micro-aspiration. Acute exacerbation of ILD (AE-ILD) has a relatively high mortality rate [1]. Management is mainly based on palliation of symptoms, treating hypoxemia and reversible etiologies of respiratory failure, if present.

Infection with the newly identified novel severe acute respiratory syndrome coronavirus 2 (SARS-CoV-2) causes various phenotypic variation of coronavirus disease (COVID-19). The spectrum of symptomatic respiratory illness varies from mild to critical cases that require mechanical ventilation and ICU care [2]. Advanced age, smoking, hypertension, diabetes mellitus, chronic lung disease, cancer, chronic kidney disease and obesity are risk factors for severe illness and mortality [3,4]. Due to the disruption of the regular healthcare system, the pandemic had an indirect impact on the health of people with chronic diseases. The major concerns for patients with preexisting ILD during the COVID-19 pandemic were restricted access to physicians, uncertainties regarding use of steroids and difficulty in follow-up [5]. Post-COVID-19 fibrosis resulting in functional deficit and long-term sequelae is an added nightmare for both the patients of ILD who survived COVID-19 infection and for the treating physician [6].

## Materials and methods

This was a retrospective observational study carried out in a tertiary care hospital of western India. Reverse transcription polymerase chain reaction (RT-PCR)-confirmed COVID-19 patients with pre-existing ILD admitted to the hospital in the period from May 1, 2020 to April 30, 2021 were enrolled in the study. Records of the patients were retrieved from the hospital electronic database. These included demographic information, presenting complaints, co-morbidities, type of ILD, CT thorax findings at the time diagnosis, treatment history for ILD (steroid, anti-fibrotics, immunosuppressants), baseline spirometry, laboratory and arterial blood gases parameters at admission, mode of oxygen therapy, need for mechanical ventilation, CT severity score on admission [7], treatment received and clinical outcomes. Laboratory parameters included complete hemogram, procalcitonin, high-sensitivity C-reactive protein (hs-CRP), D-dimer, ferritin and interleukin 6 (IL-6). CT severity score was obtained by summing the score of individual lobes on CT chest, total score was out of 25 [7].

Patients admitted to the hospital were classified as mild, moderate, severe and critically severe according to COVID-19 diagnosis and treatment protocol by the World Health Organization (WHO). Mild case was defined as having mild clinical symptoms without any imaging findings of pneumonia. Moderate case was defined as having fever, respiratory symptoms, and imaging findings of pneumonia. Severe case was defined if any of the following was present: a) Respiratory distress, respiratory rate > 30/min b) Oxygen saturation (SpO2) <93% at rest or C) Partial pressure of oxygen/fractional inspired oxygen (PaO2/FiO2) < 300 mm Hg. Critical case was defined if any of the following was present a) Respiratory failure requiring mechanical ventilation b) Shock c) Extrapulmonary organ failure or need of an intensive care unit admission [8]. 

The primary outcome was mortality among the COVID-19 patients with preexisting ILD during the course of hospital admission. The patients who were discharged from hospital were included in the survivor group. The secondary outcome was to study the factors associated with mortality in these patients. The study was approved by the All India Institute of Medical Sciences Institutional Ethics Committee (AIIMS/IEC/2020/3231). 

The data were summarized and analyzed using Statistical Package for Social Sciences (SPSS) version 23 (IBM Corp., Armonk, NY, USA). Quantitative data were presented as mean and standard deviation (mean ± S.D) if normally distributed or as median and interquartile range (median ± IQR) if non-normally distributed. Univariate analysis was performed to find relation between various predetermined risk factors for mortality. Risk factors with significant p values (p<0.05) on univariate analysis were subjected to multivariate logistic regression analysis to determine independent risk factors and the odds ratio (OR) with 95% confidence interval (CI) were calculated. Receiver operating characteristic (ROC) curve was obtained for independent risk factors and the area under curve (AUC) was calculated. 

## Results

**Figure 1:**
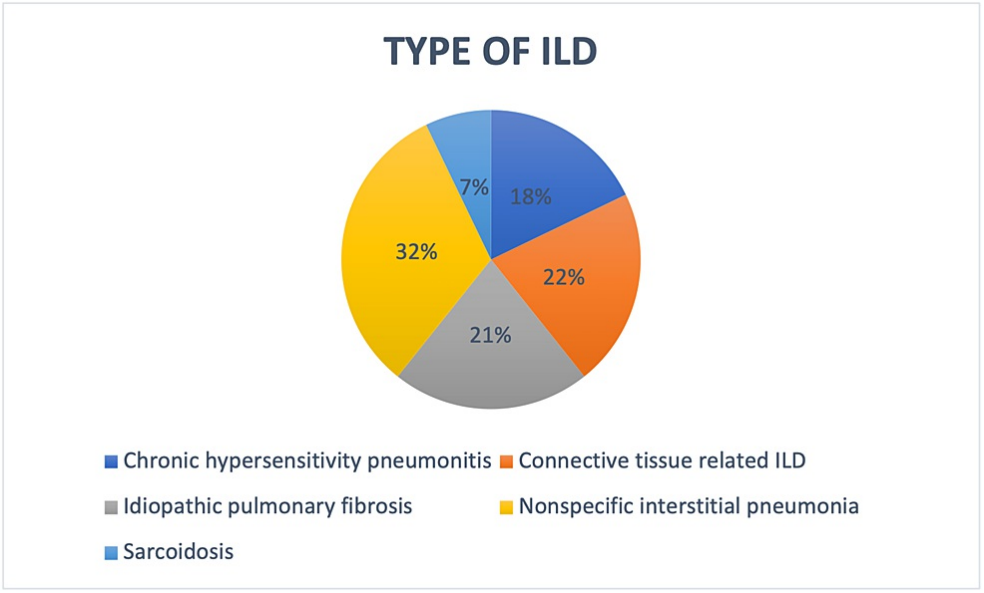
Showing type of ILD ILD: interstitial lung disease

Between May 1, 2020 and April 30, 2021, 30 patients with preexisting ILD were diagnosed with COVID-19 infection and admitted in the study center. Two patients were excluded from the study because of lack of complete data. A total of 28 patients were included in the study. Ten patients expired during the course of admission and 18 were discharged from the hospital during the study period. The demographics of the patients are shown in Table 1. The mean age of the patients was 52.03 ± 10.00 years. Majority of the patients were males 20 (71.4%). The most common pattern of ILD was nonspecific interstitial pneumonia (NSIP) present in nine patients (32.1%), followed by connective tissue-related ILD in six cases (21.4%), idiopathic pulmonary fibrosis in six cases (21.4%), fibrotic/chronic hypersensitivity pneumonitis in five cases (17.8%) and sarcoidosis in two cases (7.1%) (Figure 1). The diagnosis of ILD was on basis of Multidisciplinary Discussion (MDD) in the past. Diabetes mellitus was the most common comorbidity, seen in 13 (46.4%) patients. Ten patients were former smokers and six had a history of alcohol intake. Out of 28 patients, 14 had received antifibrotics in the past, 10 patients had received pirfenidone and four had received nintedanib. Oral corticosteroid was the most common immunosuppressant received by all patients in the past, followed by azathioprine and mycophenolate mofetil. At the time of diagnosis, on spirometry, mean forced vital capacity (FVC%) was 59.64 ± 9.86 and diffusing capacity for carbon monoxide (DLCO%) was 51.43 ± 11.99.

**Table 1:** Demographics, comorbidities, type of ILD, previous medication, lung function tests of patients of ILD with COVID-19 infection ILD: interstitial lung disease, COPD: chronic obstructive pulmonary disease, FVC: forced vital capacity, DLCO: diffusing capacity for carbon monoxide

Parameters	Total	ALIVE	DEAD	P value
Total number (n)	28	18	10	
Age ( Mean ± SD)	52.03 ± 10.00	51.33 ± 8.92	53.23 ± 11.60	0.59
Sex n (%)				
Male	20 (71.4)	12 (66.66)	8 (80.0)	0.46
Female	8 (28.6)	6 (33.3)	2 (20.0)	
Smoking n (%)	10 (35.7)	5 (27.8)	5 (50.0)	0.62
Alcohol n (%)	6(21.4)	4 (22.2)	2 (20.0)	0.82
Co-morbidities n (%)				
Hypertension	10(35.7)	6 (33.3)	4 (40.0)	0.72
Diabetes mellitus	13(46.4)	6 (33.3)	7 (70.0)	0.07
Coronary artery disease	6(21.4)	2 (11.1)	4(40.0)	0.09
COPD	4(14.2)	2 (11.1)	2 (20.0)	0.9
Asthma	1 (3.5)	1 (5.6)	0	0.9
Chronic kidney disease	1 (3.5)	1 (5.6)	0	0.92
Type of ILD n (%)				
Fibrotic hypersensitivity pneumonitis	5 (17.8)	4 (22.2)	1 (10.0)	0.54
Connective tissue related ILD	6 (21.4)	2 (11.1)	4 (40.0)	
Idiopathic pulmonary fibrosis	6 (21.4)	4 (22.2)	2 (20.0)	
Nonspecific interstitial pneumonia	9 (32.1)	6 (33.3)	3 (30.0)	
Sarcoidosis	2 (7.1)	2 (11.1)	0 (0.0)	
Pattern on CT scan at diagnosis of ILD n (%)				
Honeycombing	12 (42.9)	4 (22.2)	8 (80.0)	0.76
Ground glass appearance	18 (64.3)	8 (44.4)	10 (100)	
Septal thickening	14 (50.0)	8 (44.4)	6 (60.0)	
Tractional bronchiectasis	11 (39.3)	4 (22.2)	7 (70.0)	
Lymphadenopathy	4 (14.3)	2 (11.1)	2 (20.0)	
Medications received in past n (%)				
Corticosteroids	28 (100)	18 (100)	10 (100)	0.62
Pirfenidone	10 (35.7)	6 (33.3)	4 (40.0)	0.53
Nintedanib	4 (14.2)	2 (11.1)	2 (20.0)	0.56
Other immunosuppressants	5 (17.8)	3 (16.7)	2 (20.0)	0.39
Spirometry and DLCO%				
FVC% (Mean ± SD)	59.64 ± 9.86	61.44 ± 9.68	56.4 ± 9.35	0.2
DLCO% (Mean ± SD)	51.43 ± 11.99	52.55 ± 13.19	49.4 ± 9.12	0.508

Contrast-enhanced computed tomography (CECT) chest was done in all patients. All patients required oxygen supplementation either with low-flow oxygen delivery system like nasal prong or face mask or non-rebreather mask, high flow nasal cannula (HFNC), non-invasive or invasive mechanical ventilation. Prior to supplemental oxygenation, the mean PaO_2_/FiO_2_ at admission was 189.03 ± 52.43. Twenty-four patients were started on low-flow oxygen delivery system, eight improved without the need of high-flow oxygen delivery. A total of 16 patients in study population required oxygen via HFNC out of which eight improved, the other eight eventually required mechanical ventilation but none survived. Two patients in critical group on admission were started with mechanical ventilation. None could be successfully extubated from ventilator. Extracorporeal membrane oxygenation (ECMO) was not used in any patient. All patients received antibiotics, steroids and anti-coagulation. Remdesivir was given to 26 patients, out of which 16 survived and the other 10 expired. Plasma therapy was given to two but none survived. Patients on antifibrotic therapy on admission were continued on medication, if otherwise contraindicated like hepatic injury. 

On comparing the parameters associated with increased mortality, there was no effect of age, gender, comorbidities, type of ILD, CT features on diagnosis, use of corticosteroids and antifibrotics in the past on mortality. The FVC% was lower in patients within the non-survivor group (50.57 ± 8.38 vs 60.88 ± 9.94, p=0.03), than those who were alive but it was not significant. The difference in DLCO% between the two groups was also not significant (50.57 ± 10.50 vs 52.2 ± 13.99, p=0.76). 

Among the lab parameters between the two groups, there was no statistical difference among the two groups in terms of hematological parameters such as hemoglobin, total leucocyte count, platelet count, procalcitonin, hs-CRP, or ferritin. However, the patients in the non-survivor group had a significantly higher mean levels of IL-6 (256 ± 54.99 vs 155.55 ± 87.44, p=0.014), and D-dimer (3.209 ± 1.61 vs 1.58 ± 1.45, p=0.01), as compared to the survivor group (Table 2). 

**Table 2:** Clinical course, vitals and lab parameters of patients of ILD with COVID-19 on admission ILD: interstitial lung disease, SBP: systolic blood pressure, DBP: diastolic blood pressure, Hb: haemoglobin, TLC: total leucocyte count, IL-6: interleukin-6, hsCRP: high-sensitivity C-reactive protein # CT severity score was calculated out of 25

	Total	Alive	Dead	P value
Total no (n)	28	18	10	
Symptoms, n (%)				
Fever	26 (92.8)	16 (88.9)	10 (100)	0.86
Shortness of breath	28 (100)	18 (100)	10 (100)	0.54
Cough	21 (75.0)	13 (72.2)	8 (80.0)	0.45
Severity of presentation, n (%)				
Mild	4 (14.3)	4 (22.2)	0	0.94
Moderate	0	0	0	
Severe	22 (78.6)	14 (77.8)	8 (80.0)	
Critical	2 (7.14)	0	2 (20.0)	
Vitals (Mean ± SD)				
Spo2%	76.64 ± 5.09	77.22 ± 5.49	75.66 ± 4.07	0.42
Respiratory rate	27.21 ± 4.90	26.33 ± 5.17	28.82 ± 3.89	0.21
Heart rate	101.68 ± 16.21	97.39 ± 13.66	109.52 ± 17.47	0.07
SBP	131.93 ± 15.0	125.881 ± 2.10	139.711 ± 5.55	0.09
DBP	84.621 ± 6.97	82.0 ± 8.30	88 ± 13.61	0.33
Lab parameters (Mean ± SD)				
Hb(gm%)	12.28 ± 1.30	12.23 ± 1.09	12.38 ± 1.59	0.775
TLC (per ul)	10485 ± 3737	9452 ± 3827	12346 ± 2712	0.06
Platelet count (10^5^/ul)	1.71 ± 0.68	1.77 ± 0.02	1.68 ± 0.78	0.87
Procalcitonin	0.61 ± 1.06	0.38 ± 0.35	1.04 ± 1.63	0.24
hsCRP (mg/L)	77.4 ± 69	60.16 ± 63.24	99.23 ± 68..17	0.15
IL-6 (pg/mL)	191.42 ± 91.17	155.55 ± 87.44	256 ± 54.99	0.014
Ferritin (ug/L)	317 ± 205.8	306.22 ± 379.52	327.1 ± 389.9	0.9
D-dimer (mg/L)	2.16 ± 1.70	1.58 ± 1.45	3.209 ± 1.61	0.01
Pa0_2_/FiO_2_ on admission	189.03 ± 52.43	202.2 ± 37.70	181.33 ± 59.16	0.39
CT Severity score on admission #	17.8 ± 3.4	14.22 ± 3.78	21.5 ± 1.20	0.9
Duration of hospital stay	14.25 ±1 0.95	19.11 ± 10.93	10.5 ± 9.03	0.08

**Figure 2:**
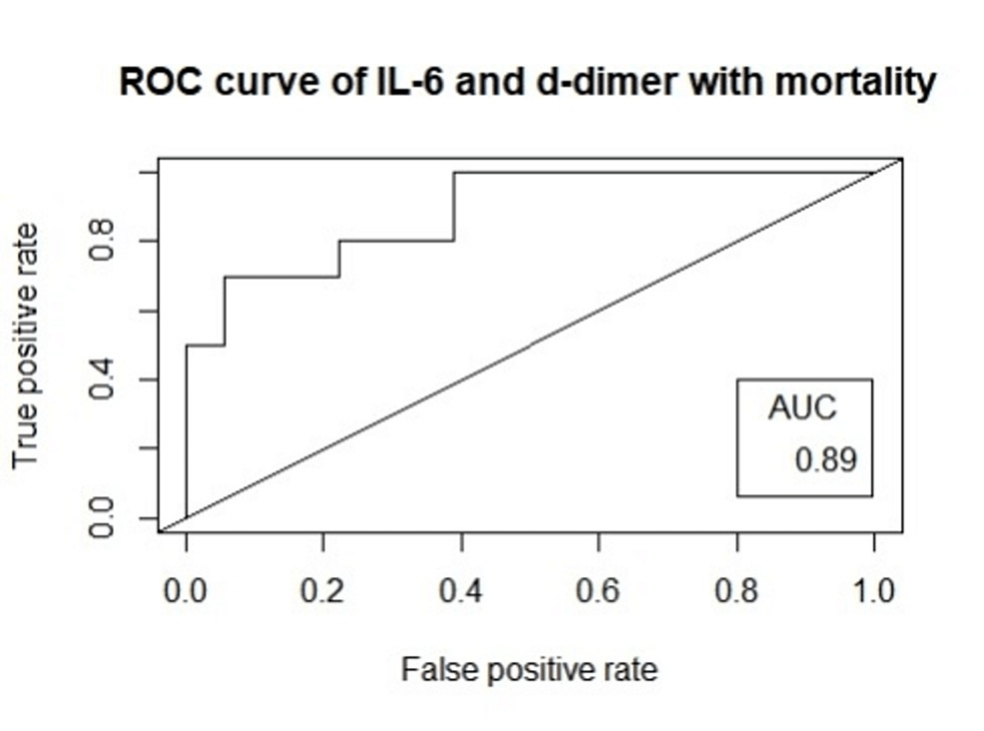
Showing ROC curve of IL-6 and D-dimer with mortality IL-6: interleukin 6, ROC: receiver operating characteristic, AUC: area under curve

The data were analyzed using logistic regression to identify the factors associated with poor outcomes in patients. On multivariate analysis, the significant parameters were IL-6, p=0.02, OR=1.020 (1.006-1.043) and D-dimer, p=0.04, OR=2.14 (5.55-1.14). Figure 2 shows ROC curve with IL-6 and D-dimer as prognostic model of mortality. AUC was 0.89.

## Discussion

In this study, we analyzed the demographics, clinico-radiological characteristics, hospital course and clinical outcome of patients with preexisting ILD who were admitted with COVID-19 infection in the hospital. The majority (22/28) of the patients had severe COVID-19 disease on presentation. It could be because patients with mild COVID-19 infection were managed at home with symptomatic treatment.

The overall mortality of ILD patients with COVID-19 in our study was 35.7%. This is in line with a multicenter study by Drake et al., where the mortality rate among hospitalized ILD patients with COVID-19 was 49% while in another study from US, the mortality rate was 33% [9,10]. It is in variance to an Indian study by Chauhan et al., which showed lower mortality (19.2%) among patients hospitalized with COVID-19 infection compared to our study [11]. One of the reasons for high mortality among hospitalized COVID-19 patients with pre-existing ILD could be the difficulty to differentiate manifestations of superimposed severe COVID-19 infection from acute exacerbation or progression of underlying ILD. The radiological resemblance and absence of serum biomarkers to differentiate the two pose a challenge for the treating physician. High dose corticosteroids with or without immunosuppressants, and broad-spectrum antibiotics are being used for management of AE-ILD [12]. The management of severe COVID-19 infection has been on different line, with use of corticosteroids, anti-viral agents like remdesivir.

Among the inflammatory markers studied, IL-6 emerged as an independent risk factor of mortality. IL-6 is an inflammatory cytokine produced during a cytokine storm and represents a heightened Th1-type inflammatory response. JAKs (Janus kinases) and STAT3 (signal transducer and activator of transcription 3) mediate downstream signal transduction of IL-6. This leads to a cascade of reaction at the cellular level leading to systemic hyperinflammation, releasing other cytokines and chemokines which contribute to vascular hyperpermeability, leakiness, hypotension, and pulmonary dysfunction [13].

In a study, IL-6 in COVID-19 patients emerged superior to other biomarkers like CRP, ferritin, and liver enzymes for predicting death, with an optimal cut-off of 200 pg·L−1, respectively. The mean IL-6 values in the non-survivor group in our study was 256 ± 54 pg.L-1 [14]. Studies have also looked into the role of IL-6 in the clinical outcome of patients with ILD without COVID-19 infection. In the study by Lauretis et al., IL-6 was a prognostic marker of mortality within 30 months in patients with IPF and systemic sclerosis-induced ILD [15]. IL-6 has also been proposed to cause lung fibrosis, it skews the inflammation towards Th17 type and also results in suppression of Th1 response and differentiation of CD4+ cells into a profibrotic Th2 type which promotes lung fibrosis [16]. Saito et al. studied the role of IL-6 in the pathogenesis of lung fibrosis by injecting bleomycin in mice. They found that an important role of IL-6 in the development of bleomycin-induced lung inflammation and fibrosis, possibly through the upregulation of TGF-beta1 and CCL3 [17].

In recent studies, the bidirectional relationship between inflammation and thrombosis is well established. “Thromboinflammation” results due to loss of normal anti-inflammatory and anti-thrombotic activity of endothelial cells resulting in widespread coagulation and inflammation [18]. The study by Ranucci et al. [19] showed a correlation between fibrinogen and IL-6 in COVID -19 patients which attests to a milieu of inflammatory thrombosis. D-dimer is used as a marker of the procoagulant state and clinical outcomes in COVID-19 patients. Yao et al. [20] carried out a meta-analysis to look at clinical factors to predict mortality in COVID-19 patients and found that raised D-dimer levels were strongly associated with higher mortality risk (OR=10.5). In another meta-analysis by Gungor et al. [21], 16 studies were incorporated, D-dimer levels above the ULN had a risk ratio of 1.82 for mortality. The analysis also found that the weighted mean difference of D-dimer levels between the surviving and non-surviving groups was 5.32 mg/L, 95% CI: 3.90-6.73. In the study by Ishikawa et al., the elevated D-dimer level has been described as a risk factor for development of acute exacerbation of ILD (OR=10.46) [22]. In our study also, D-dimer emerged as an independent risk factor of mortality (OR=2.14) with the mean D-dimer values in the non-survivor and survivor groups being 3.2 and 1.58 mg/L respectively with an optimal cut off point of 1.76 mg/L.

The dysregulated activation of the coagulation cascade has been linked to the pathophysiology of several acute and chronic lung diseases. Factor Xa, a proteinase of the coagulation pathway, has been described as the driver for fibrotic response to lung injury [23]. COVID-19 has also been described as hypercoagulable state, with the role of cross-talk between coagulation and inflammatory pathways increased risk of thrombosis leading to higher mortality [24]. The markers of coagulopathy like D-dimer can provide a prognostic insight and guide anticoagulation therapy in patients of COVID-19 with co-existent ILD.

In our study, there was no statistically significant difference in FVC% between survivor and non-survivor groups (p=0.2). Infection with COVID-19 in patients with already compromised lung function can bring about a cascade of events including a virus triggered acute exacerbation of ILD and cytokine storm. This two-pronged respiratory insult can lead to poorer clinical outcomes in ILD patients. Drake et al. in their international multicenter study found that ILD patients with COVID-19 infection with FVC (% predicted) <80%, were at a higher risk of mortality (p=0.039). In the study by Gallay et al. [25], it was found that FVC (% predicted) was a risk factor for mortality (p=0.02) in univariate analysis.

Our study showed that the spiromteric parameters are not associated with mortality, which is counterintuitive. But the elevated markers of thrombosis and inflammation represented by D-dimer and IL-6 levels were a better predictor of mortality. Studies have shown that patients of IPF with low FVC are at a higher risk of mortality following acute exacerbation [26]. But exacerbation of ILD following COVID-19 is different from the other forms of ILD exacerbation in its rapid and widespread systemic involvement. The non-significant association of FVC with mortality in our study may point to the fact that rather than spirometric variables, the dysregulated cytokine response of the body to COVID-19 is the main factor determining mortality.

The study has limitations of small sample size and retrospective nature. The mild cases of COVID-19 infection who were not admitted were not included in the study which may have impacted the mortality rate. Serial biochemical testing was not incorporated in the study which might have yielded a better prognostic model by investigating the trend of various biomarkers.

## Conclusions

COVID-19 in ILD patients has a comparatively higher mortality. Elevated D-dimer and IL-6 levels may be significant predictors of mortality in patients of ILD infected with COVID-19. These investigations can serve as sentinels for identifying ILD patients at higher risk. Although IL-6 is an inflammatory marker, the complex interplay between phenotypic characteristics of the patient and the SARS-CoV-2 virus will determine in which way the inflammatory cascade will propagate to: fibrotic or non-fibrotic.
